# Efficacy of physical therapy for the treatment of lateral epicondylitis: a meta-analysis

**DOI:** 10.1186/s12891-015-0665-4

**Published:** 2015-08-25

**Authors:** Christoph Weber, Veronika Thai, Katrin Neuheuser, Katharina Groover, Oliver Christ

**Affiliations:** Department of Psychology, TU Darmstadt, Alexanderstrasse 10, 64287 Darmstadt, Germany; DMB Die MPU Berater GmbH, Bad Nauheimerstrasse 4, 64289 Darmstadt, Germany; Justizvollzugsanstalt Darmstadt, Marienburgstrasse 74, 64297 Darmstadt, Germany; School of Applied Psychology, University of Applied Sciences and Arts NortherwesternSwitzerland, Riggenbachstrasse 16, 4600 Olten, Switzerland

## Abstract

**Background:**

Physical therapy for the treatment of lateral epicondylitis (LE) often comprises movement therapies, extracorporeal shockwave therapy (ECSWT), low level laser therapy (LLLT), low frequency electrical stimulation or pulsed electromagnetic fields. Still, only ECSWT and LLLT have been meta-analytically researched.

**Methods:**

PUBMED, EMBASE and Cochrane database were systematically searched for randomized controlled trials (RCTs). Methodological quality of each study was rated with an adapted version of the Scottish Intercollegiate Guidelines Network (SIGN) checklist. Pain reduction (the difference between treatment and control groups at the end of trials) and pain relief (the change in pain from baseline to the end of trials) were calculated with mean differences (MD) and 95 %-Confidence intervals (95 % CI).

**Results:**

One thousand one hundred thirty eight studies were identified. One thousand seventy of those did not meet inclusion criteria. After full articles were retrieved 16 studies met inclusion criteria and 12 studies reported comparable outcome variables. Analyses were conducted for overall pain relief, pain relief during maximum handgrip strength tests, and maximum handgrip strength. There were not enough studies to conduct an analysis of physical function or other outcome variables.

**Conclusions:**

Differences between treatment and control groups were larger than differences between treatments. Control group gains were 50 to 66 % as high as treatment group gains. Still, only treatment groups with their combination of therapy specific and non-therapy specific factors reliably met criteria for clinical relevance. Results are discussed with respect to stability and their potential meaning for the use of non-therapy specific agents to optimize patients’ gain.

**Electronic supplementary material:**

The online version of this article (doi:10.1186/s12891-015-0665-4) contains supplementary material, which is available to authorized users.

## Background

Lateral epicondylitis (LE) is a painful musculoskeletal condition caused by overuse. The injury of the common extensor tendon originating from the lateral epicondyle is better known as tennis elbow. Both names are misleading though, since it is neither an inflammatory condition, nor does it only occur in tennis players. Other sports and jobs involving highly repetitive movements are strong contributors to the overuse-injury. It mostly affects people 40 years and older. Some studies indicate that men and women are equally affected [1], others report a higher percentage of affected women [1, 2]. The general prevalence rate ranges from 1 to 3 % per year [2]. The National Guidelines Clearinghouse [3] recommends to first inform patients about the condition and to instruct them further to avoid aggravation [3]. The first pharmacological approach is to prescribe nonsteroidal anti-inflammatory drugs (NSAIDs). Also injection therapies for lateral epicondylitis are suggested. In a systematic review [4] the effects of prolotherapy, polidocanol, whole blood and platelet-rich plasma on lateral epicondylitis were measured. Strong pilot-level evidence was found but all studies were limited by small sample size. Newer studies showed small to none effects of injection therapies on pain and disability [5, 6]. In general, treatments like splinting, stretching and strengthening exercises, soft tissue mobilisation and acupuncture are recommended [3].

Research on physical treatments for LE has not yet proven superiority of one specific approach. A meta-analysis by the Cochrane Collaboration [2] found little to no superiority of shock wave therapy over placebo and Bjordal et al. [7] found only short term effects of low level laser therapy (LLLT) over placebo. Both meta-analyses focused on one form of physical treatment.

The aim of this study was to meta-analyse the empirical evidence for physical treatments for LE and give practitioners an estimate of what benefits patients might expect from various treatments, both based on treatment specific and non-specific agents. Outcome differences between baseline and end-of-treatment were calculated for treatment and control groups as well as differences between treatment and control groups at end-of-treatment. Heterogeneity is discussed for each of these analyses.

## Methods

### Searching

We searched PUBMED, EMBASE and the Cochrane Database until April 2012 using medical subject headings related to epicondylitis when possible. The Search Key included the following key words: tendinoses, tendinosis, tendinitides, tendinitis, tendonitides, tendonitis, tendinopathy, epicondylalgia, epicondylitides, epicondylitis, tennis elbow. Further we hand-searched references of systematic reviews until April 2012 for additional studies. To identify grey literature we searched clinicaltrials.gov for registered RCTs on physical therapy for LE patients. Limits were set to randomized controlled trials with adults (18 years and older) and language restrictions were set to languages spoken by the authors (i.e., English and German).

### Selection

Studies were eligible if they investigated a physical therapy intervention in comparison to a waiting-list control group, treatment as usual control group or sham-control group. If a study investigated a combination of therapy modalities (e.g., extra corporeal shockwave therapy in combination with manual therapy) the control group would have to match one of the therapy modalities (e.g., only extra corporeal shock wave therapy or only manual therapy). Orthoses, acupuncture, massage regimens, surgery, pharmacological treatments and psychotherapy were not included into the meta-analysis. Patients had to be diagnosed with LE. All outcomes were considered for inclusion as long as at least three studies used the same outcome measurement. Studies had to report mean, standard deviation and number of participants at baseline and at the end of treatment.

Study design was limited to RCTs, and each group under investigation had to consist of 10 or more patients.

### Validity assessment

Four raters in groups of two independently rated the included studies, using an adapted form of the SIGN Checklist for RCTs. The checklist consisted of eight items evaluating the key question, randomization procedure, blinding, comparability of treatment and control groups with respect to baseline measurements, study procedure and additional therapies, validity of outcome measurements, dropout rates and the use of intention-to-treat analysis. The rating was conducted in three steps. Differences in step one were resolved by exchanging citations between raters, followed by re-rating. Differences in step 2 were resolved by discussion. Inter-rater reliability was calculated by Cohen’s κ for each rating step and with eight items per study.

Items were assessed either as “well”, “poor” or “not addressed”/“not reported”. If randomization (Item 1.2) was rated as “not addressed” or “not reported”, the study was excluded for not meeting RCT criteria. Studies in which all aspects were rated as "well", were classified as Level of Evidence (LoE) “++” for “good, very low risk of bias”. If four or more aspects were rated as”poor” or “not addressed” the study was classified as LoE “-“for “poor, high risk of bias”. Studies were also rated as LoE “poor, high risk of bias” if the comparability of groups with respect to study procedures was deemed compromised (Item 1.6). Similar, if neither intention-to-treat analysis was performed (Item 1.9) nor adequate blinding measures were employed (Item 1.4), the study was rated as LoE “poor, high risk of bias”. All other studies were classified as LoE “+” “fair, low risk of bias”.

### Data extraction

The following data were extracted from each study: means and standard deviations of pain intensity, Disabilities of the Arm, Shoulder and Hand (DASH) function score, maximum handgrip strength in kg, pain during maximum handgrip strength test, group size, type of treatment, control group intervention, treatment duration, treatment frequency, assessment schedules and time since diagnosis of LE. All pain scales were transformed linearly to a 0–100 point scale. For scales from 0 to y: *transformed MEAN* = *MEAN* × (100 ÷ *y*). For scales from 1 to y: *transformed MEAN* = (*MEAN* − 1) × (100 ÷ (*y* − 1)). Standard deviations were transformed as follows: *transformed SD* = *SD* × (100 ÷ *y*) for scales from 0 to y; *transformed SD* = *SD* × (100 ÷ (*y* − 1)) for scales from 1 to y. All hand grip strength scales were transformed into kg. If no minimum and maximum duration of illness was reported, mean plus/minus two standard deviations was used to estimate the interval which should include about 95 % of participants.

Data were extracted by two independent investigators, differences were solved by discussion. Since there was only one LLLT study and one ECSWT study which reported DASH scores, no further analysis was conducted for physical function.

### Quantitative data synthesis

Effect sizes were calculated by mean differences (MD). Given standard errors were transformed into standard deviations. No authors were contacted for missing data. Statistical heterogeneity was assessed by I^2^ = [(Q – df)/Q] × 100 %, where Q is the chi-squared statistic and df is its degrees of freedom. I^2^ describes the percentage of the effect estimates variability which can be attributed to heterogeneity. Since effect sizes of studies testing against waiting-list (WLC) or treatment as usual control groups tend to be higher than those testing against sham-control or active control groups, studies were split into three sub-groups; 1) waiting-list or treatment as usual control groups, 2) sham-control groups, and 3) studies which compared a combination of two treatments to the single application of one of those treatments. Publication bias was assessed by Egger’s regression intercept using Comprehensive-Meta-Analysis Software (CMA Software).

### Statistical methods and outcomes

Results are reported as MD [95 % CI] (I^2^). Mean Difference (95 % Confidence Interval] (Heterogeneity); with (s.) showing statistical significance and (n.s.) showing non significance. Two types of MDs are being reported. MDs between treatment and control groups are indicated as “difference between treatment and control groups”. MDs between baseline and end-of-treatment are indicated as “difference from baseline”.

## Results

### Trial flow

**Fig. 1:**
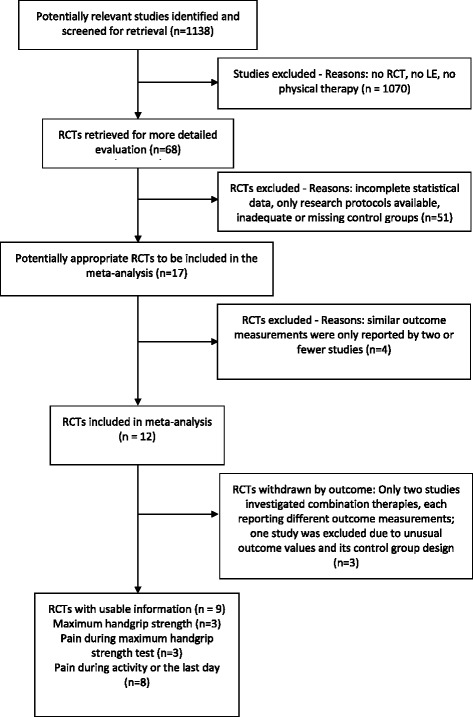
Flow diagram of the article selection process

Figure 1 shows a flow diagram of the selection processes. One thousand one hundred thirty eight studies were identified. One thousand seventy of those did not meet inclusion criteria. The remaining 68 were retrieved as full text articles and checked for inclusion and exclusion criteria once again. Seventeen studies met all criteria and were considered for quantitative synthesis. Twelve of those reported comparable outcome measures. Since only two studies [8, 9] investigated a combination of therapies, each reporting different outcome measurements, neither study was included in the meta-analyses. Only one study used a WLC design and therefore was excluded [10]. The remaining nine studies were included in the analysis; three investigated LLLT, four ECSWT, one low frequency electrical stimulation and one pulsed electromagnetic field therapy (PEMF). There were not enough comparable studies to evaluate any other treatment (Table 1).

### Study characteristics

#### Quantitative data synthesis

Sixteen studies were included in the rating procedure [8–23]. One study was rated as LoE “++” [11], 7 studies were rated as LoE “+” [10, 15, 17–21] and 8 studies were rated as LoE “-“[8, 9, 12–14, 16, 22, 23]. Cohen’s κ was calculated to assess inter-rater reliability for each rating step κ_step1_ = 0.46; κ_step2_ = 0.83; κ_step3_ = 1.

In the end, five analyses could be conducted; the first on the effect of physical therapy (ECSWT, LLLT, low frequency electrical stimulation and PEMF) on pain; the second on the effect of extracorporeal shockwave therapy (ECSWT) on pain; the third on the effect of non-ECSWT treatments (LLLT, low frequency electrical stimulation and PEMF) on pain; the fourth on the effect of LLLT on pain during maximum handgrip strength tests, and the fifth on the effect of physical therapy treatments (LLLT and ECSWT) on maximum handgrip strength. The analysis on the effect of physical therapy on physical functioning was not conducted due to the heterogeneity of measurement instruments. Two studies reported DASH (sports/music, work) scores, one DASH function, one an adapted patient specific function scale, and one the upper extremeties function scale. The authors considered these scales too heterogeneous to combine.

Review Manager Software (RevMan 5) by the Cochrane Collaboration was used to conduct the five analyses.

All reported pain outcomes were transformed to a 0–100 scale and all grip strength outcomes to kg.

#### Overall pain ECSWT, LLLT, low frequency electrical stimulation and PEMF

Outcomes used were pain during the last 24 h, pain during activity, pain during Thomsen Test, pain during day and night, and pain at isometric testing.

**Fig. 2:**
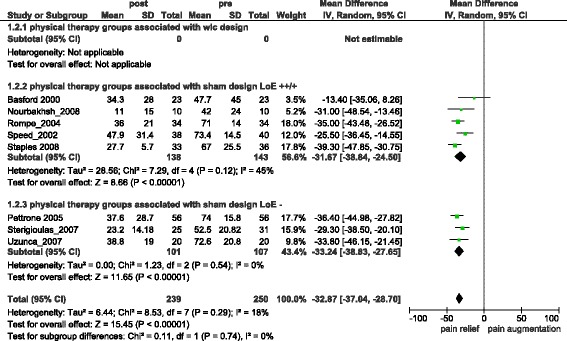
Overall pain relief in treatment groups

**Fig. 3:**
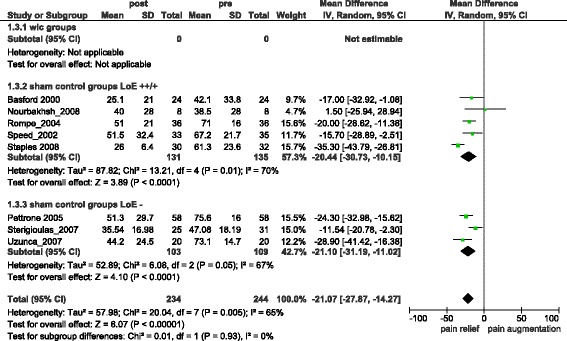
Overall pain relief in sham-groups

Combined Pain relief in treatment groups (difference from baseline) was −32.87 [95 % CI = −37.04, −28.70] (I^2^ = 18 %) (s.) (Fig. 2), with only one study [24] reporting pain relief below 25. Combined Sham-control groups reported −21.07 [95 % CI = −27.87, −14.27] (I^2^ = 65 %) (s.) (Fig. 3) units of pain relief (difference from baseline). Comparing pain intensity outcomes of treatment and control groups at the end of treatment resulted in −7.50 [95 % CI = −14.94, −0.07] (I^2^ = 78 %) (s.) (Additional file 1) units difference in pain reduction.

#### Overall pain ECSWT

**Fig. 4:**
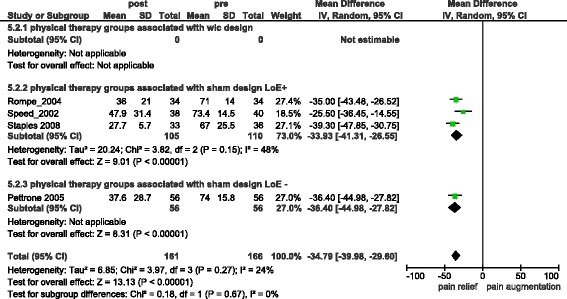
Overall pain relief in ECSWT groups

**Fig. 5:**
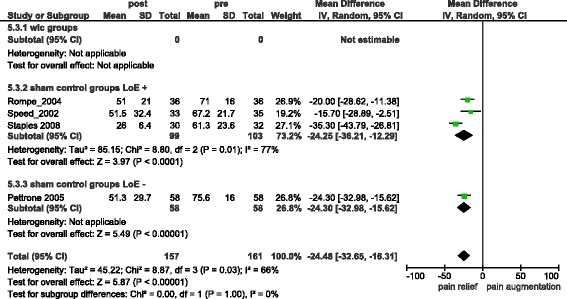
Overall pain relief in sham-ECSWT groups

If only ECSWT studies were analysed combined treatment groups reported −34.79 [95 % CI = −39.98, −29.60] (I^2^ = 24 %) (s.) (Fig. 4) units of pain relief (difference from baseline). Combined Control groups in ECSWT studies reported −24.48 [95 % CI = −32.65, −16.31] (I^2^ = 66 %) (s.) (Fig. 5) units of pain relief (difference from baseline). Comparing pain intensity between ECSWT and control groups at the end of studies resulted in a statistically non significant pain reduction of −7.20 [95 % CI = −17.44, 3.04] (I^2^ = 82 %) (n.s.) (Additional file 2). Three of these four studies were of high methodological quality reporting a combined pain reduction of 5.13 [95 % CI = −16.71, 6.46] (I^2^ = 82 %) (n.s.) (difference between treatment and control groups).

Only two studies remained for a LLLT sub-group analysis. Thus, no effect size calculations were conducted.

#### Overall pain LLLT, low frequency electrical stimulation and PEMF

**Fig. 6:**
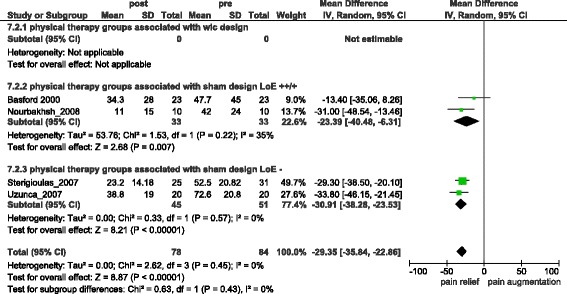
Overall pain relief in Non-ECSWT treatment groups

**Fig. 7:**
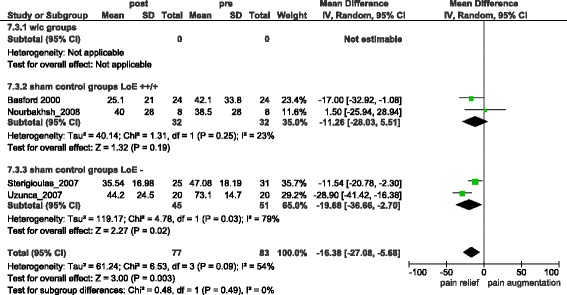
Overall pain relief in Non-ECSWT sham-groups

Two LLLT studies, one low frequency electrical stimulation study and one PEMF study reported sufficient data to be analysed. Combined Non-ECSWT treatment groups gained −29.35 [95 % CI = −35.84, −22.86] (I^2^ = 0 %) (s.) (Fig. 6) units of pain relief (difference from baseline). The respective combined control groups gained −16.38 [95 % CI = −27.08, −5.68] (I^2^ = 54 %) (s.) (Fig. 7) (difference from baseline). Comparing treatment and control groups at the end of trials resulted in a pain reduction of −8.12 [95 % CI = −20.83, 4.60] (I^2^ = 71 %) (n.s.) (Additional file 3).

#### Pain during maximum handgrip strength tests

Three studies reported data on pain during maximum handgrip strength tests, all investigating LLLT. Combined treatment groups gained −19.16 [95 % CI = −25.20, −13.11] (I^2^ = 0 %) (s.) (Additional file 4) units of pain relief (difference from baseline). Control groups gained −2.58 [95 % CI = −11.69, 6.52] (I^2^ = 33 %) (n.s.) (Additional file 5) units of pain relief (difference from baseline). Difference in pain intensity between treatment and control groups at end of treatment was −7.92 [95 % CI = −22.65, 6.81] (I^2^ = 79 %) (n.s.) (Additional file 6).

### Physical function

Only two studies remained for a physical function analysis. Thus, no effect size calculations were conducted.

### Maximum handgrip strength

Three studies reported maximum grip strength, two investigating LLLT and one investigating ECSWT. Treatment groups had mean maximum handgrip strength gain of 6.47 kg [95 % CI = 3.68, 9.26] (I^2^ = 0 %) (s.) (Additional file 7) (difference from baseline). Control groups had a mean maximum handgrip strength gain of 2.81 kg [95 % CI = −1.25, 6.88] (I^2^ = 0 %) (n.s.) (Additional file 8) (difference from baseline). Comparison between treatment and control groups at the end of studies showed a MD of 3.47 kg [95 % CI = 0.17, 6.76] (I^2^ = 0 %) (s.) (Additional file 9) in favour of treatment groups. Since there was only one ECSWT and two LLLT studies, no sub-group analyses were conducted.

### Risk of bias across studies

Egger’s regression intercept showed no significant small study effects for overall pain reduction t(6) = 1.83, p = 0.25; overall pain reduction in ECSWT t(2) = 0.24; p = 0.83; overall pain reduction in non-ECSWT t(2) = 1.32; p = 0.32; pain reduction during maximum handgrip strength tests t(1) = 2.28; p = 0,26 and maximum handgrip strength t(1) = 0,47; p = 0,72.

## Discussion

### Summary of key findings

Two other meta-analyses have analyzed the effects of either ECSWT [2] or LLLT [7] on LE. This meta-analysis differs from its predecessors in two major aspects. One, it tried to investigate a wide variety of physical treatments, both in changes from baseline and differences between treatment and control groups at the end of treatment. Two, only completely published data was used and no authors were contacted for further data.

All in all, treatment groups had between 29 and 35 units and control groups between 16 and 25 units of pain relief. Differences between treatment and control groups at the end of treatment were generally low, ranging only from 7 to 9 units on a 0–100 scale. Of five comparisons between treatment and placebo groups only one, the combined analysis of ECSWT and non-ECSWT studies, showed statistically significant results. This finding should be interpreted with utmost reluctance, since neither ECSWT studies alone, nor Non-ECSWT studies alone showed statistically significant differences between treatment and placebo groups. With rather large pain relief scores in both, treatment and placebo groups, and only small differences between treatment and placebo groups it can be concluded that a large portion of therapy effects are attributable to contextual factors.

These findings resemble those of Buchbinder et al. [2] who found that ECSWT is no more effective than placebo. For pain at rest they report a MD (pain out of 100) of −9.42 [95 % CI = −20.7, 1.86].

Bjordal et al. [7] analyzed 7 studies of LLLT for the treatment of LE. In contrast to Bjordal et al. [7] this meta-analysis identified only 2 LLLT studies which both, met inclusion criteria and published sufficient data for meta-analysis. This meta-analysis did not include six studies which were included in Buchbinder et al. [2]. Five studies were excluded due to not reported standard deviations [9, 13, 25–27], one was not included since the underlying data is not published [28–35].

Since there were no authors contacted for this meta-analysis a lower number of studies was to be expected. Due to the small number of studies this meta-analysis offers no interpretation concerning the effectiveness of LLLT in the treatment for LE. Bjordal et al. [7] concluded that LLLT was safe and effective and that it acted in a dose dependent manner.

Pain relief during maximum handgrip strength tests was generally lower than overall pain relief. Treatment groups had a mean pain relief of 19 units on a 0–100 scale and control groups had about 3 units. Still, differences in comparisons between those groups at the end of treatment resulted in only 8 units of pain reduction on a 0–100 scale, which might partly come from a shift of weights in this analysis. Treatment groups’ maximum handgrip strength improved by 6 kg while control groups improved by 3 kg. The mean difference between treatment and control groups at the end of treatment was 3 kg.

Both, Buchbinder et al. [2] and Bjordal et al. [7] explicitly state the need for further research. Buchbinder et al. [2] especially criticize “a lack of uniformity in both the timing of follow up and the outcomes that were measured”. This meta-analysis found the same methodological heterogeneity. As can be seen in Table 1, treatment duration, treatment intensity, symptom duration, times of measurement and reported outcomes vary largely between studies.

**Table 1:** Studies considered for inclusion

Article	Author	Year	Reported outcomes	Treatment duration	Times of measurements	Symptom duration	Treatment
[11]	Basford et al.	2000	^a^pain in last 24 h maximal tenderness on palpation	3S/W for 4 Ws	2 Ws, 4 Ws, follow-up	1–17 Ms	LLLT vs placebo
			^a^maximum grip strength pinch strength				
			^a^pain with grasp pain with pinch				
[12]	Bisset et al.	2009	reaction time	8S over 6 Ws	6, 52 Ws	6–89 Ms	Physical therapy vs WLC
[13]	Haake et al.	2002	side effects	1S/W over? Ws	?	1–99 Ms	ECSWT vs placebo
[7]	Ho et al.	2007	mechanical pain threshold	10S over 3 Ws	1, 2, 3 Ws, follow-up 3 Ws	3–15 Ms	Microcurrent & exercise vs exercise
			^b^pain-free handgrip strength				
			^b^maximum handgrip strength pain during max grip				
[14]	Lam et al.	2007	mechanical pain threshold	3S/W for 3 Ws	Session 5, 9 & 3 Ws after completion	1–9 Ms	LLLT vs placebo
			^a^maximum grip strength				
			^a^pain after grip strength test				
			Disability score				
			DASH (sports/music)				
			DASH (work)				
[9]	Martinez-Silvestrini et al.	2005	pain free grip strength	Daily for 6 Ws	6 Ws	3+ Ms	Stretching vs stretching & concentric vs stretching & eccentric strengthening
			^b^VAS pain				
			PRFEQ				
			^b^dash function				
[15]	Nourbakhsh et al.	2008	^c^maximum grip strength	6S over 2–3 Ws	Post treatment, follow-up	6–60 Ms	Low frequency electrical stimulation vs placebo
			^a^pain intensity last 24 h functional level (adapted patient specific function scale) limited activity due to pain				
[10]	Peterson et al.	2011	pain MVC	1S/D exercise regimen over 3 Ms	1, 2, 3 Ms	3+ Ms	Exercise vs WLC
			pain MME				
			^b^muscular strength activity score well-being complaint score				
[16]	Pettrone et al.	2005	^a^pain during Thomsen test function activity score overall impression	1S/W over 3 Ws	1, 4, 8, 12 Ws; 6, 12 Ms only reported at 12 Ws	6+ Ms	ECSWT vs placebo
			^a^grip strength adverse events				
[17]	Rompe et al.	1996	night pain	1S/W over 3 Ws	3, 6, 24 Ws after last application	12+ Ms	ECSWT vs sham
			resting pain				
			pressure pain				
			Thomsen test				
			finger extension				
			chair test				
			^c^grip strength (Mucha and Wannske)				
[18]	Rompe et al.	2001	pressure pain	1S/W over 3 Ws	12 Ws, 12 Ms	12–208 Ms	ECSWT vs ECSWT + manual therapy
			Thomsen test resisted finger extension chair test				
[19]	Rompe et al.	2004	^a^pain during Thomsen test	1S/W over 3 Ws	3, 12 Ms	12+ Ms	ECSWT vs sham
			Roles and Maudsley score upper extremity function scale				
			^b^dynanometer test				
[20]	Speed et al.	2002	^a^Pain (day and night) night pain 50 % improvement from baseline	1S/M for 3 Ms	1; 2; 3 Ms	3–42 Ms	ECSWT vs placebo
[21]	Staples et al.	2008	^a^overall pain index function index (0–100 VAS) pain-free function index dash function (0–100) dash sport (0–100) dash work (0–100) pain free grip ratio	1S/W over 3 Ws	6 Ws; 3, 6 Ms	6–520 Ws	ECSWT vs sham
			^b^max grip strength SF-36 role limitation physical bodily pain general health vitality social function role limitation emotional mental health				
			health transition problem elicitation technique PET global health				
[22]	Sterigioulas et al.	2007	pain at rest	2S/W over first 4 Ws 1S/W over second 4 Ws	8 Ws, 8 Ws after end of treatment	5 Ws-12 Ys	LLLT & exercise vs placebo and exercise
			pain at palpation				
			^a^pain on isometric testing pain during middle finger test				
			^a^pain during grip strength test				
[47]	Öken et al.	2008	Grip strength	5S/W over two weeks	2 Ws, 4 Ws after end of treatment	1–24 Ms	Ultrasound & hot pack vs LLLT & hot pack vs brace control group
			Pain severety				
			Global assessment of improvement				
[23]	Uzunca et al.	2007	resting pain	5S/W over 3 Ws	3 Ws, 3 Ms	1–11 Ms	Pulsed electromagnetic field (PEMF) vs sham PEMF
			^a^activity pain				
			night pain				
			pain during resisted wrist				
			dorsiflexion pain during resisted forearm supination algometric pain threshold				

*W* week, *M* month, *S* Session

^a^Outcome used in meta-analysis

^b^Not included due to control group design

^c^Not included since the authors could not assuredly establish a method to transform data into kg

## Conclusions

Treatment groups showed more homogeneous outcomes than we expected from the differing treatment modalities (I^2^ = 18 %). The mean pain relief amounted to 32.9 units in treatment groups and to 21.1 units in control groups. The difference between treatment and control groups in mean pain relief amounted to 11.8 units on a 0–100 scale. Thus, control groups gained about 2/3 of treatment groups’ overall pain relief. Differences between ECSWT (34.8 units of pain relief) and non-ECSWT studies (30.4 units of pain relief) only amounted to 4 units. This means that the difference between treatments seems to be lower than the difference between treatments and their respective control groups. If further studies produced similar results this might indicate that the decision which physical therapy treatment to use (ECSWT, LLLTlow frequency electrical stimulation or PEMF) might not be as important as maximizing non-treatment specific effects.

During physical therapy patients do not only benefit from the treatment itself, e.g., the pharmacological effect of a drug or the physical effect of a laser therapy, but also from non-treatment specific agents, the so called sham-effects, placebo-effects or contextual effects [36]. Patients’ pain relief thus results from a combination of treatment specific agents and non-treatment specific agents. Important non-specific agents can be e.g., spontaneous remission, expectancy, motivation, conditioning and other psychosocial agents [36].

With the combination of contextual and therapy-specific factors about 95 % of patients in treatment groups gained between 28 and 38 units of pain relief on a 0–100 scale, compared to 14 to 28 units in control groups and by contextual effects, only.

The difference between treatment and placebo groups at the end of treatment was rather low. Still, only treatment groups with their combination of specific and unspecific agents managed to rather reliably reach clinically important pain relief of more than 22 units on a 0–100 scale [37]. Patients in sham groups with their purely unspecific agents only gained clinically relevant pain relief in less than 50 % of cases.

### Limitations

Altogether, for overall pain 473 patients were analyzed, for pain during maximum handgrip strength test 136 patients and for maximum handgrip strength 193 patients. These numbers are much lower than those reported of patient collectives, studied e.g., in pharmaceutical trials for WHO I (non-opioid analgesics) or WHO II (weak opioids) analgesics which regularly evaluate over 100 patients per group per study [33, 38–46]. In the overall pain analysis 318 of 473 patients were treated with ECSWT, 97 with LLLT, 18 with low frequency electrical stimulation, and 40 with pulsed electromagnetic field therapy. Thus, ECSWT results might be relatively stable while non-ECSWT results might change, even with only a few new studies.

Patients varied largely in their duration of symptoms, making it impossible to differentiate between studies with only acute or only chronic LE patients. Minimum symptom duration varied between 4 weeks and 12 months, maximum duration between 9 months and 17 years, with several studies not reporting a cut-off point at all.

While some studies investigated treatment effects as early as after the last treatment session, some studies let several weeks or months pass before measuring post treatment effects. Even though follow-up investigations help understand the long-term effects of a therapy, a prolonged period of time between the end of a treatment and the assessment of its effectiveness may distort results. Especially changes in patients’ activities or therapy regimen, as well as social context may influence trial results.

Another distorting factor in this meta-analysis was the rather large difference in treatment durations and sessions per week. Studies went on over time periods of at least three weeks to a maximum of three months. During this time treatments were applied a minimum of once per month to a maximum of five sessions per week. Thus, study effects were achieved with largely differing efforts.

Still overall pain relief (I^2^ = 18 %), pain relief during maximum handgrip strength tests (I^2^ = 0 %) and increase in maximum handgrip strength (I^2^ = 0 %) in treatment groups effects were mostly homogeneous. Only overall pain relief in control groups (I^2^ = 65 %) showed great heterogeneity and pain relief during maximum handgrip strength tests (I^2^ = 33 %) showed medium to low heterogeneity. Thus contributing to rather large heterogeneity in the end of treatment comparisons of overall pain (I^2^ = 78 %) and pain during maximum handgrip strength tests (I^2^ = 79 %).

## Additional files

Additional file 1:
**Overall pain reduction for physical therapy groups.** (PDF 332 kb) Additional file 2:
**Overall pain reduction in ECSWT groups.** (PDF 267 kb) Additional file 3:
**Pain reduction in Non-ECSWT groups.** (PDF 274 kb) Additional file 4:
**Pain during grip strength test relief in LLLT groups.** (PDF 240 kb) Additional file 5:
**Pain during maximum grip strength test relief in LLLT-sham groups.** (PDF 243 kb) Additional file 6:
**Pain during maximum handgrip strength test reduction in LLLT groups.** (PDF 245 kb) Additional file 7:
**Maximum grip strength gain in treatment groups (LLLT and ECSWT).** (PDF 233 kb) Additional file 8:
**Maximum grip strength gain in sham-groups (associated with LLLT and ECSWT).** (PDF 231 kb) Additional file 9:
**Differences between treatment and sham-groups in maximum handgrip strength at the end of treatment (LLLT and ECSWT).** (PDF 235 kb)

## Abbreviations

CI: Confidence interval
DASH: Disabilities of the arm, shoulder and hand function score
ECSWT: Extracorporeal shockwave therapy
Kg: Kilogram
LE: Lateral epicondylitis
LLLT: Low level laser therapy
LoE: Level of evidence
MD: Mean difference
MVC: Maximum voluntary contraction
MME: Maximum muscle elongation
NSAID: Non-steroidal anti-inflammatory drugs
PEMF: Pulsed electromagnetic field therapy
PET: Problem elicitation technique
PRFEQ: Patient-related forearm evaluation questionnaire
RCT: Randomized controlled trial
SF36: Short form 36 health survey
SIGN: Scottish Intercollegiate Guidelines Network
VAS: Visual analogue scale
WLC: Waiting-list control group
WHO: World health organization
