# Supplementary figures and images for: Efficacy of physical therapy for the treatment of lateral epicondylitis: a meta-analysis
Source: BMC Musculoskelet Disord. 2015 Aug 25;16:223. doi: 10.1186/s12891-015-0665-4 (PMC4549077; doi:10.1186/s12891-015-0665-4)

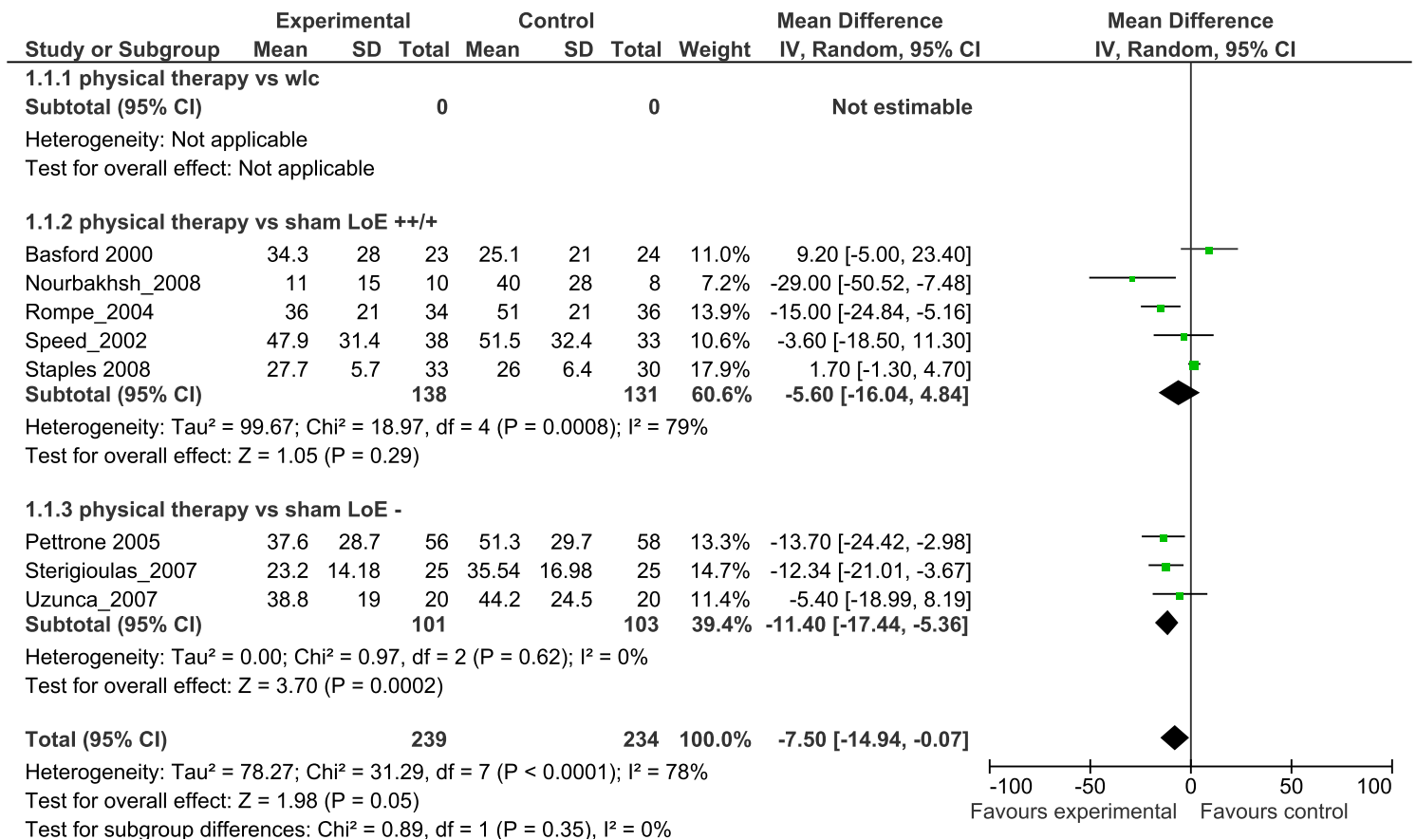

Supplement: Additional file 1: — Overall pain reduction for physical therapy groups. (PDF 332 kb) [file 12891_2015_665_MOESM1_ESM.pdf]

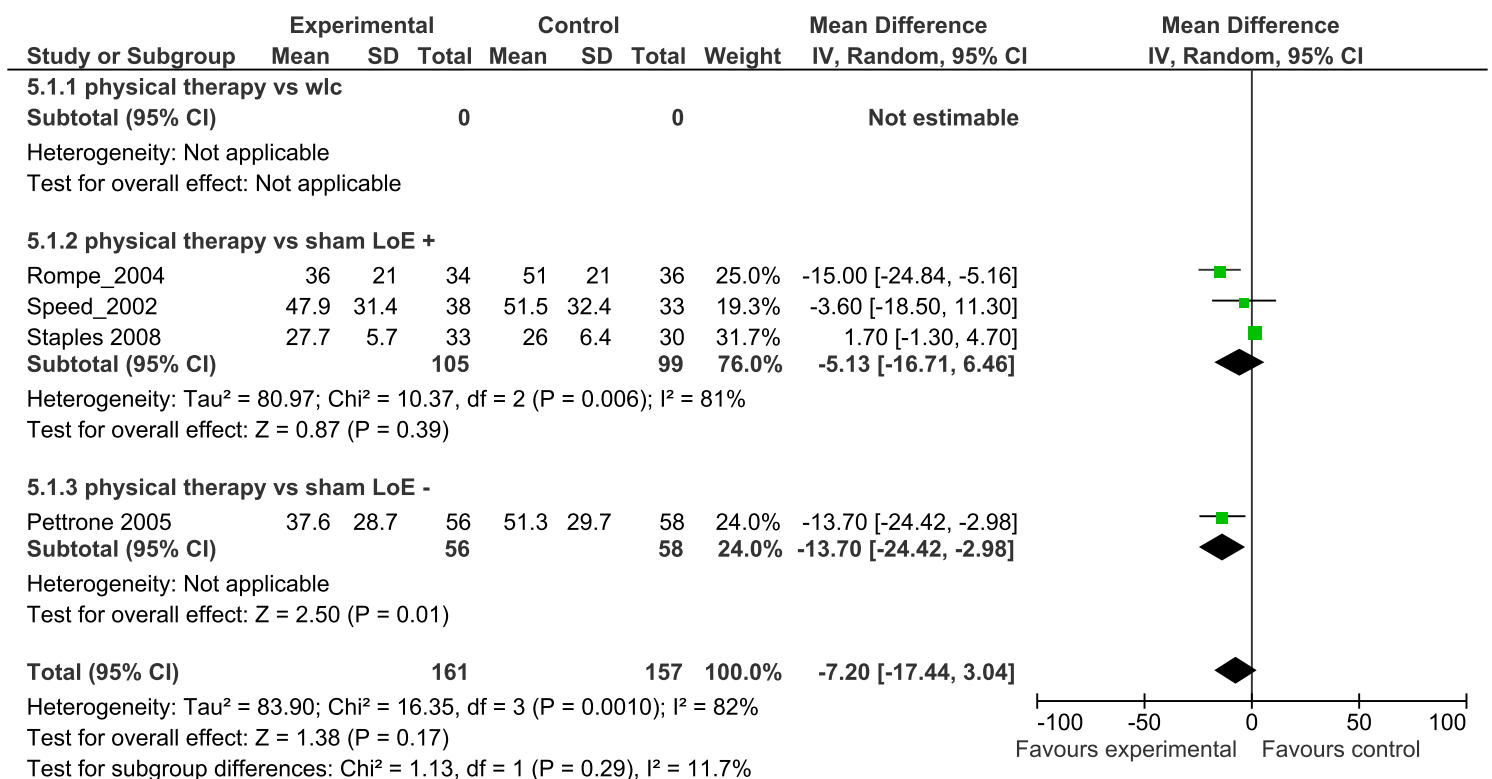

Supplement: Additional file 2: — Overall pain reduction in ECSWT groups. (PDF 267 kb) [file 12891_2015_665_MOESM2_ESM.pdf]

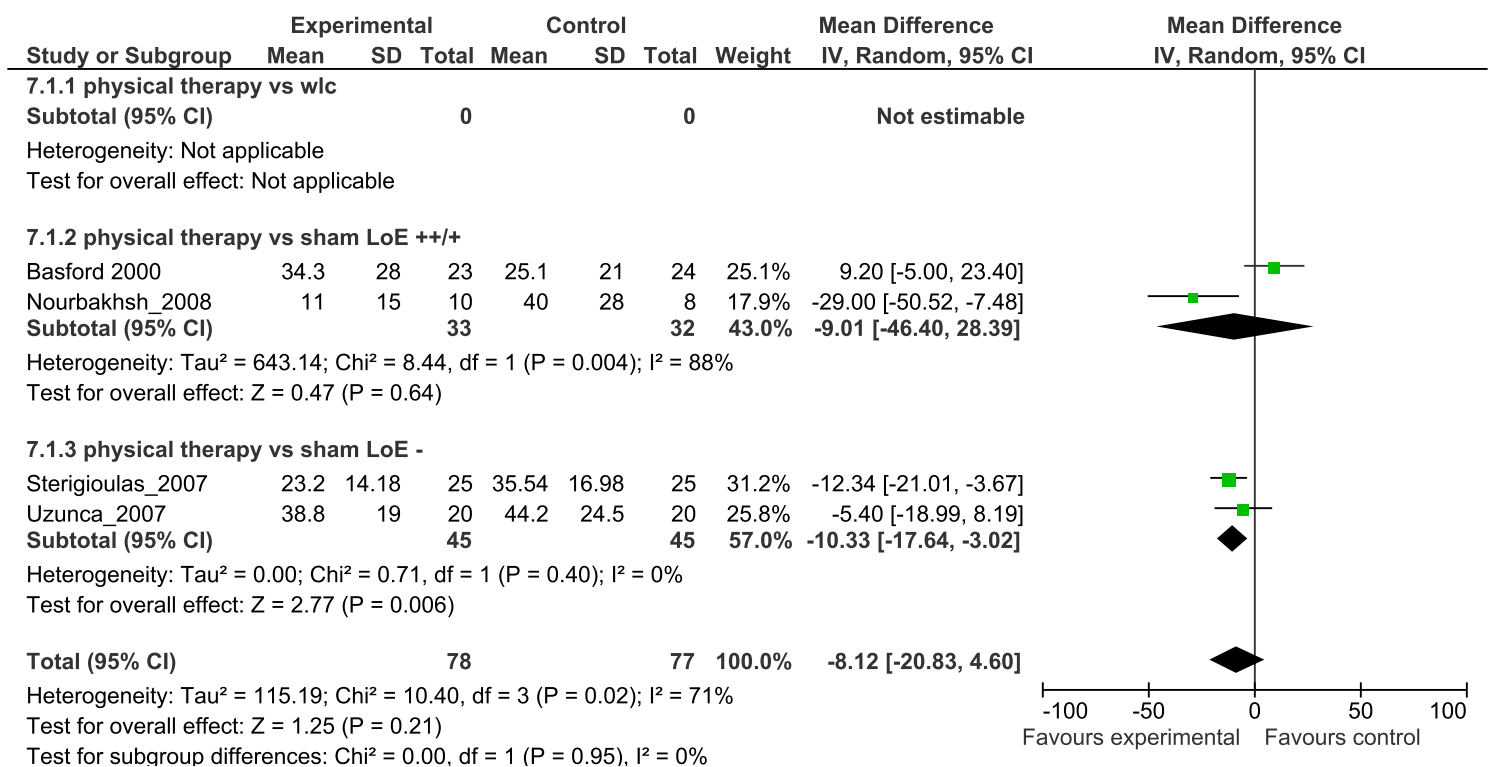

Supplement: Additional file 3: — Pain reduction in Non-ECSWT groups. (PDF 274 kb) [file 12891_2015_665_MOESM3_ESM.pdf]

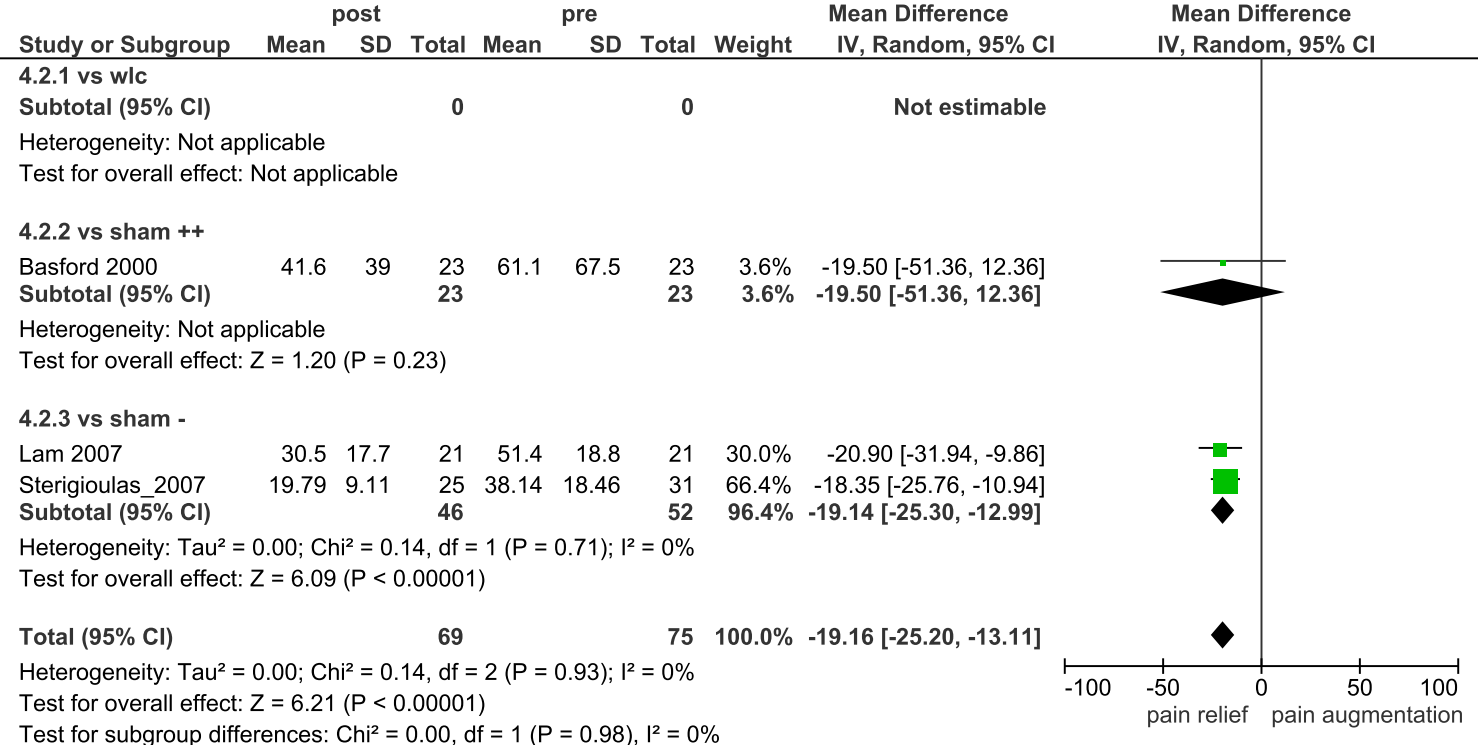

Supplement: Additional file 4: — Pain during grip strength test relief in LLLT groups. (PDF 240 kb) [file 12891_2015_665_MOESM4_ESM.pdf]

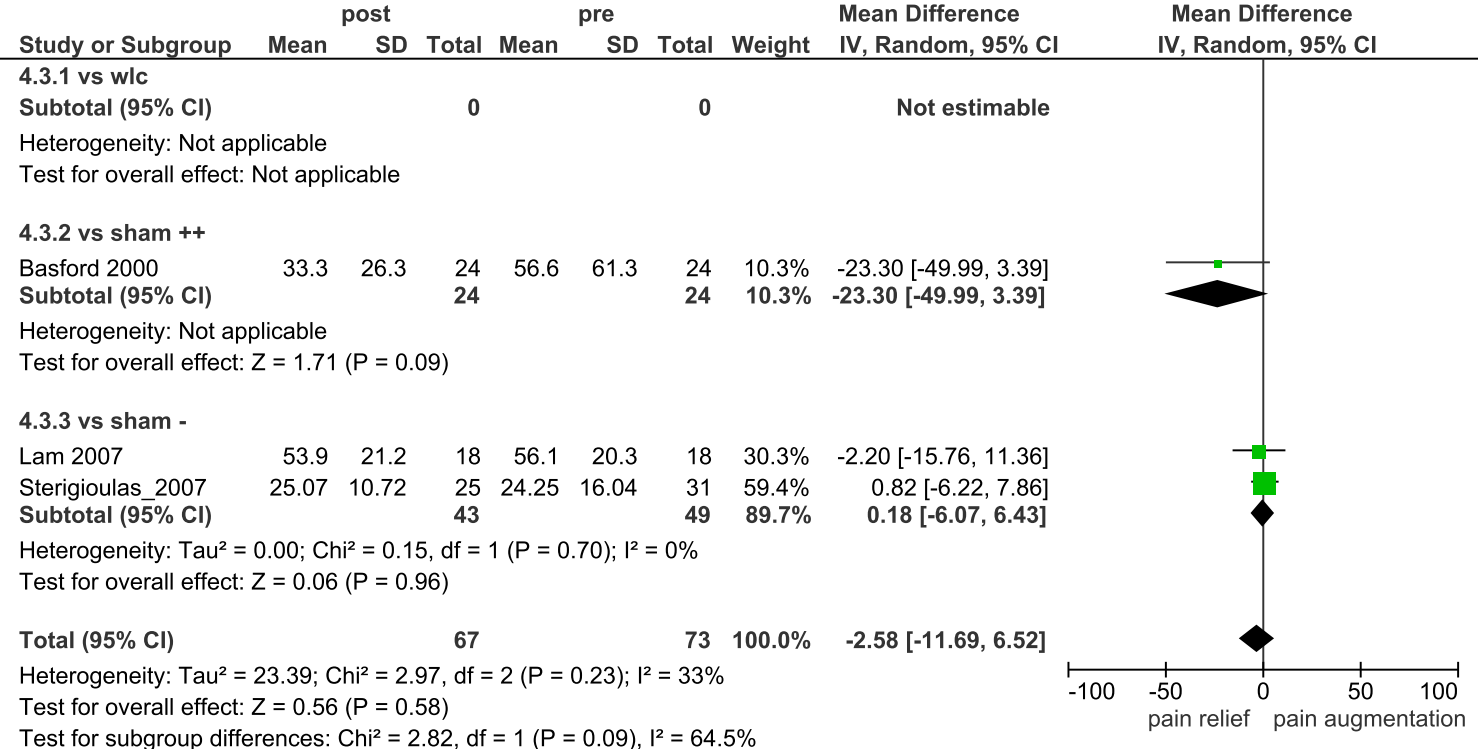

Supplement: Additional file 5: — Pain during maximum grip strength test relief in LLLT-sham groups. (PDF 243 kb) [file 12891_2015_665_MOESM5_ESM.pdf]

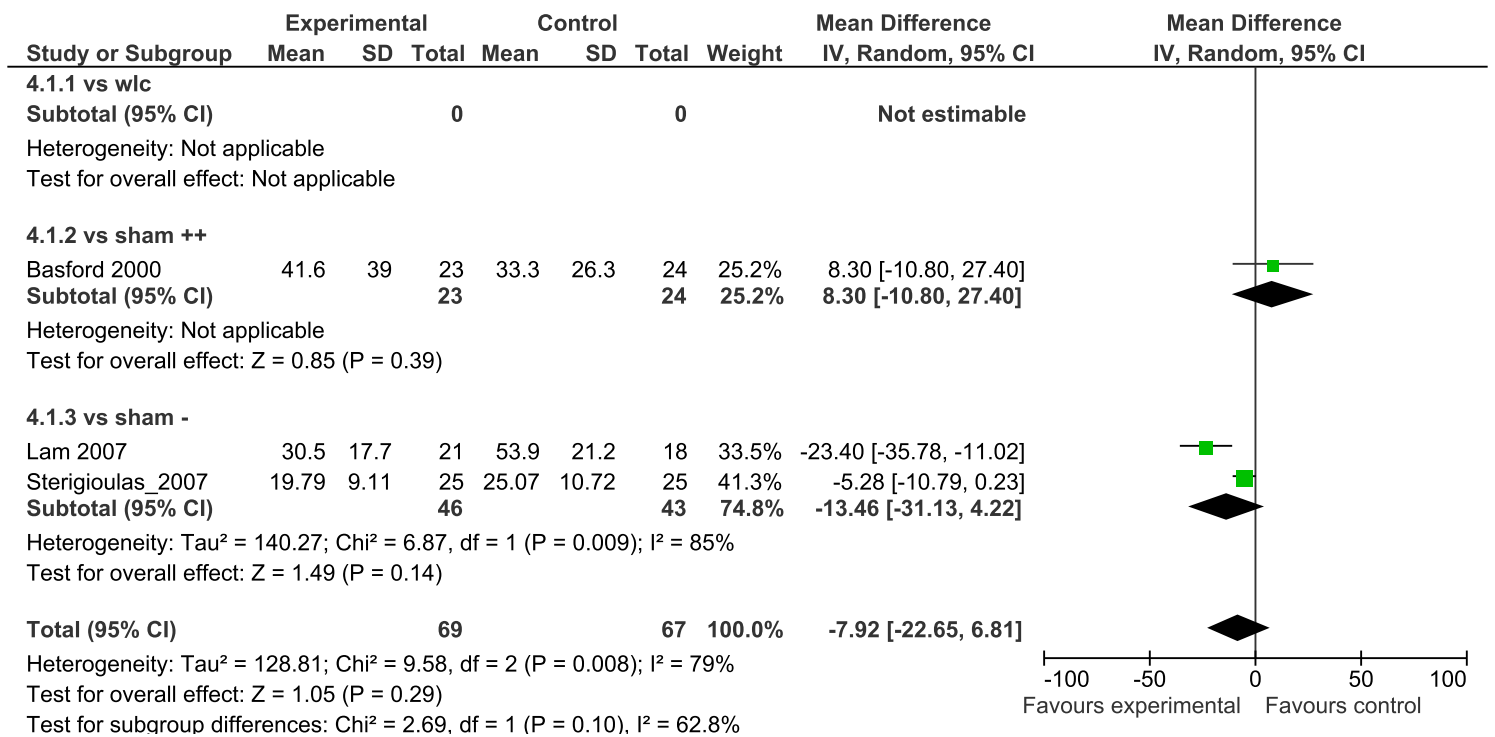

Supplement: Additional file 6: — Pain during maximum handgrip strength test reduction in LLLT groups. (PDF 245 kb) [file 12891_2015_665_MOESM6_ESM.pdf]

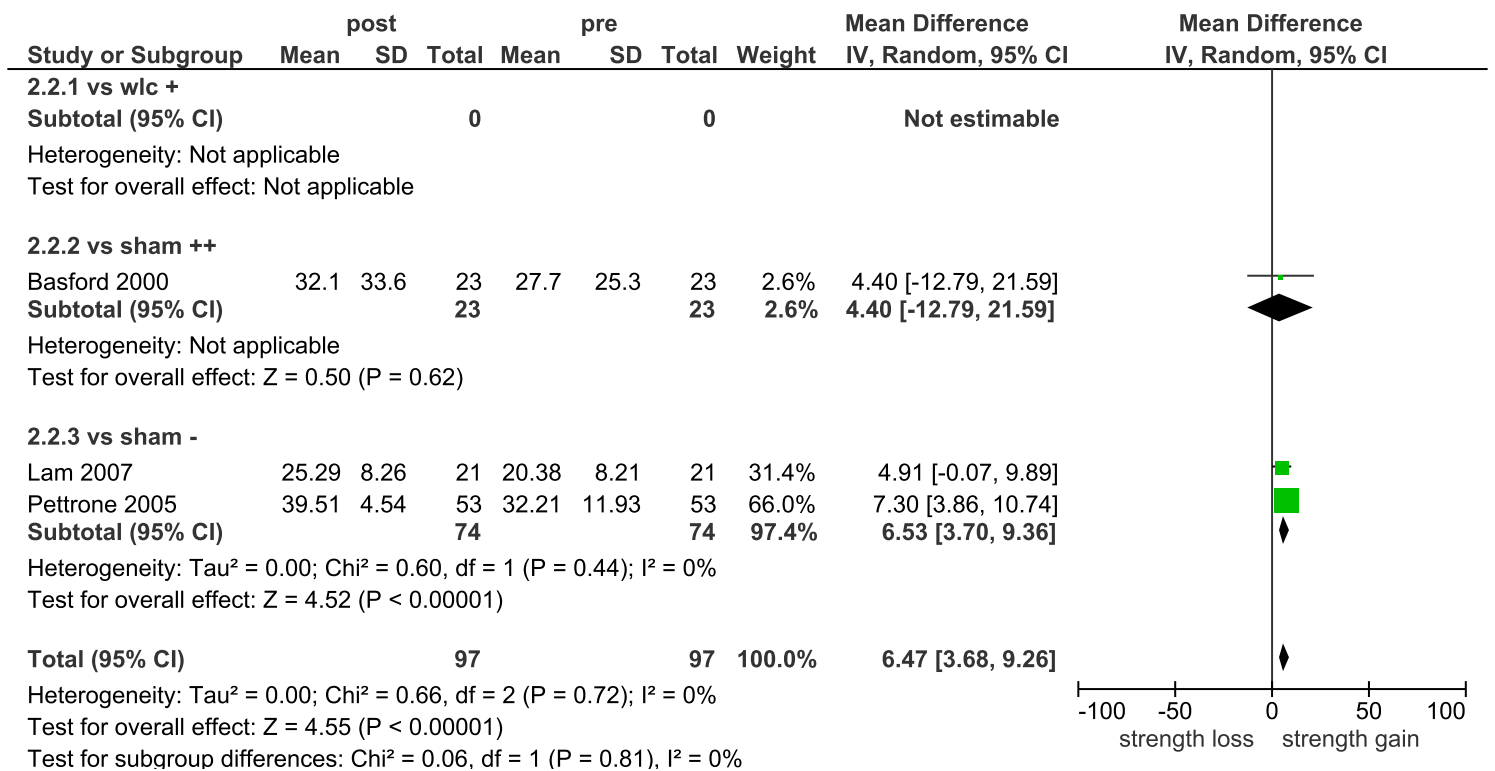

Supplement: Additional file 7: — Maximum grip strength gain in treatment groups (LLLT and ECSWT). (PDF 233 kb) [file 12891_2015_665_MOESM7_ESM.pdf]

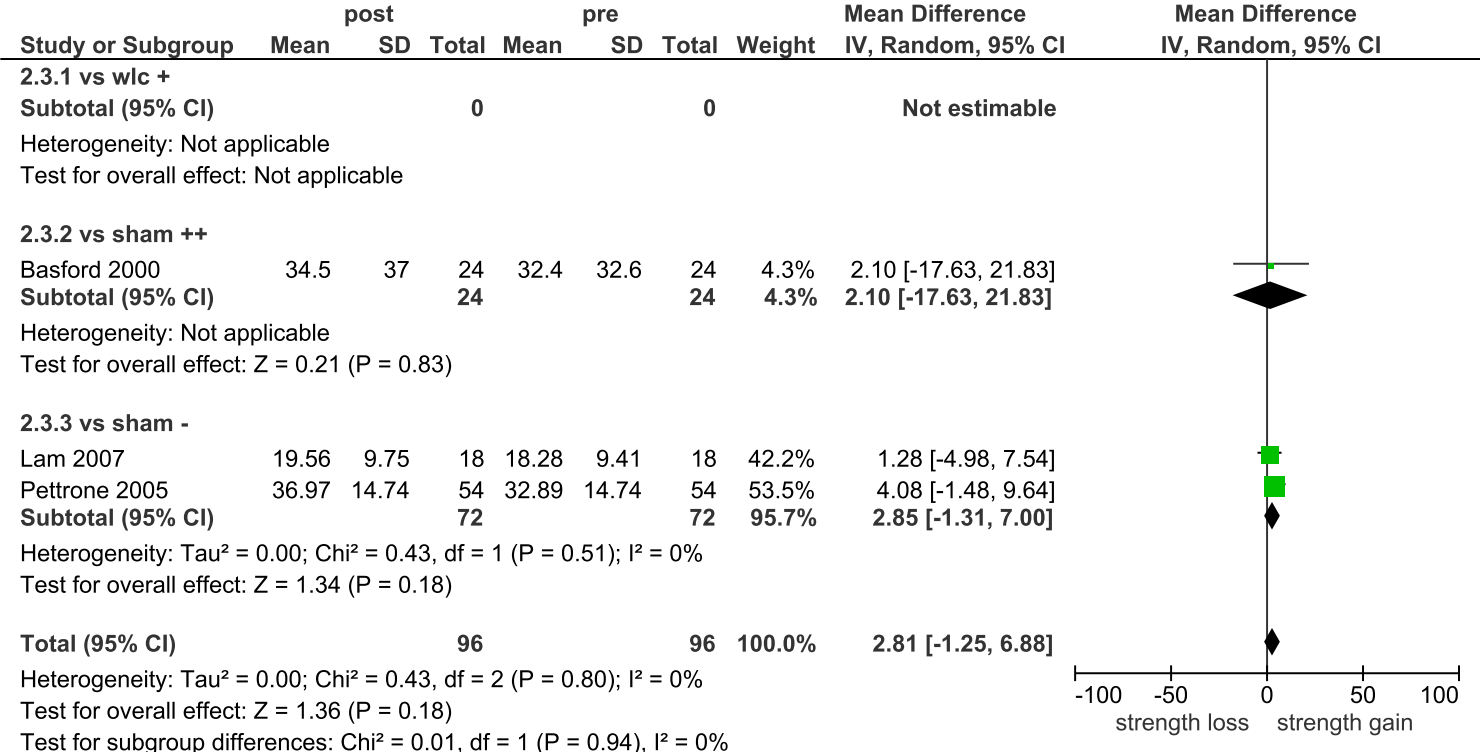

Supplement: Additional file 8: — Maximum grip strength gain in sham-groups (associated with LLLT and ECSWT). (PDF 231 kb) [file 12891_2015_665_MOESM8_ESM.pdf]

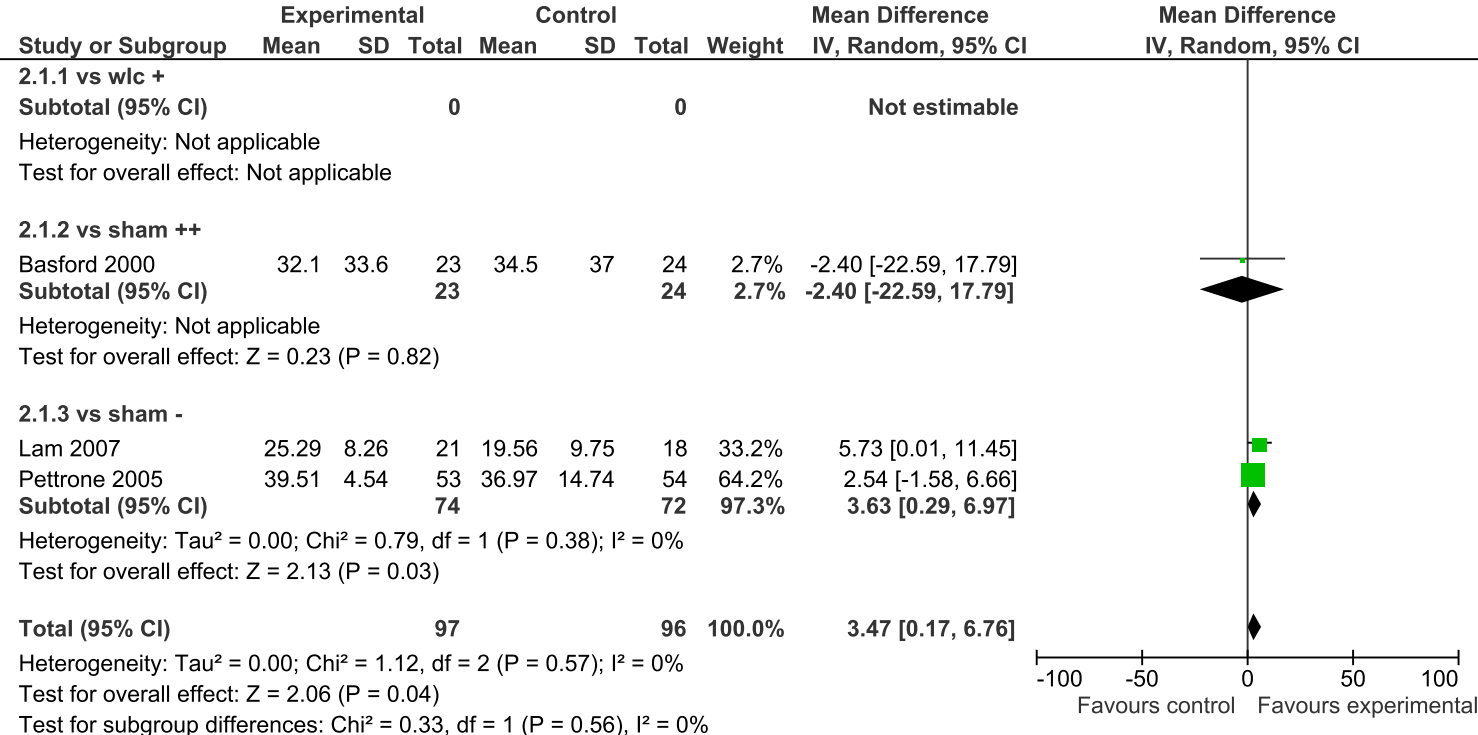

Supplement: Additional file 9: — Differences between treatment and sham-groups in maximum handgrip strength at the end of treatment (LLLT and ECSWT). (PDF 235 kb) [file 12891_2015_665_MOESM9_ESM.pdf]
